# Patient-cooperative control increases active participation of individuals with SCI during robot-aided gait training

**DOI:** 10.1186/1743-0003-7-43

**Published:** 2010-09-10

**Authors:** Alexander Duschau-Wicke, Andrea Caprez, Robert Riener

**Affiliations:** 1 Sensory-Motor Systems Lab, Institute of Robotics and Intelligent Systems, Department of Mechanical and Process Engineering, ETH Zurich, Zurich, Switzerland; 2 Spinal Cord Injury Center, University Hospital Balgrist, University of Zurich, Zurich, Switzerland; 3 Hocoma AG, Volketswil, Switzerland; 4 Institute for Human Movement Sciences, ETH Zurich, Zurich, Switzerland

## Abstract

**Background:**

Manual body weight supported treadmill training and robot-aided treadmill training are frequently used techniques for the gait rehabilitation of individuals after stroke and spinal cord injury. Current evidence suggests that robot-aided gait training may be improved by making robotic behavior more patient-cooperative. In this study, we have investigated the immediate effects of patient-cooperative versus non-cooperative robot-aided gait training on individuals with incomplete spinal cord injury (iSCI).

**Methods:**

Eleven patients with iSCI participated in a single training session with the gait rehabilitation robot Lokomat. The patients were exposed to four different training modes in random order: During both non-cooperative position control and compliant impedance control, fixed timing of movements was provided. During two variants of the patient-cooperative path control approach, free timing of movements was enabled and the robot provided only spatial guidance. The two variants of the path control approach differed in the amount of additional support, which was either individually adjusted or exaggerated. Joint angles and torques of the robot as well as muscle activity and heart rate of the patients were recorded. Kinematic variability, interaction torques, heart rate and muscle activity were compared between the different conditions.

**Results:**

Patients showed more spatial and temporal kinematic variability, reduced interaction torques, a higher increase of heart rate and more muscle activity in the patient-cooperative path control mode with individually adjusted support than in the non-cooperative position control mode. In the compliant impedance control mode, spatial kinematic variability was increased and interaction torques were reduced, but temporal kinematic variability, heart rate and muscle activity were not significantly higher than in the position control mode.

**Conclusions:**

Patient-cooperative robot-aided gait training with free timing of movements made individuals with iSCI participate more actively and with larger kinematic variability than non-cooperative, position-controlled robot-aided gait training.

## Background

Body weight supported treadmill training (BWSTT) has become a widely used rehabilitation technique for individuals with walking disabilities due to neurological disorders such as stroke and spinal cord injury [1-4].

Robotic devices have been developed to relieve physical therapists from the straineous and unergonomical burden of manual BWSTT [5]. The Lokomat (Hocoma AG, Switzerland) [6], the ReoAmbulator (Motorika, USA), and the Gait Trainer (Reha-Stim, Germany) are used in clinical practice to automate BWSTT by moving patients repetitively along pre-defined walking trajectories.

A growing body of studies shows that both manual BWSTT and robot-aided treadmill training improve gait quality [7-15]. While some of these studies found advantages of robot-aided treadmill training compared to BWSTT [9,11,14], others found conventional treadmill training to be more effective [12,13].

The studies in favor of robot-aided treadmill training focused more closely on non-ambulatory patients, while the studies reporting better outcome of conventional treadmill training included mainly ambulatory patients. These results suggest that currently, robot-aided treadmill training is most effective for severely affected, non-ambulatory patients, whereas it may not be ideal for more advanced, ambulatory patients. In contrast to these ambulatory patients, who may benefit more from other approaches like over-ground training, patients in the transition phase between being non-ambulatory and ambulatory still require much physical support during training. This situation demonstrates the need to improve current rehabilitation robots in a way that extends their spectrum of effective treatment to functionally more advanced patients. Such an improvement would allow patients to benefit from robot-aided treadmill training up to a point where they can safely and efficiently perform over-ground training. Thus, rehabilitation robots would be able to optimally support patients in their progression through their different stages of recovery.

In most of the studies mentioned above, the rehabilitation robots were controlled in a very simple way. A pre-recorded gait pattern was replayed by the robot as accurately as possible. This *position control *approach allows the patient to remain passive during the training [16] and reduces kinematic variability to a minimum [17]. However, both active participation and kinematic variability are considered as important promotors of motor learning and rehabilitation [18-23]. fMRI studies comparing training tasks with active and passive movements have shown stronger cortical activation and subsequently also more cortical reorganization leading to more effective formation of motor memory when subjects where contributing actively to the trained movements compared to being passively moved [18,19]. In a review of robotic therapy approaches based on these findings, Dromerick et al. conclude that these approaches are effective, but rigorous comparisons with traditional techniques still need to be performed [20].

Bernstein emphasized the crucial role of kinematic variability during motor learning ("repetition without repetition") based on practical experience and theoretical considerations [21]. Lewek et al. have shown that kinematic variability as introduced by conventional treadmill training improved the coordination of intralimb kinematics in ambulatory stroke patients while position-controlled robot-aided treadmill training with little kinematic variability did not [22]. Huang and Krakauer argue that from a computational motor-learning perspective, robots should ensure the successful completion of movements, allowing the adapting human nervous system to identify combinations of sensory states and their transitions associated with the motor commands required for the movements [23].

Therefore, researchers in the field of rehabilitation robotics believe that robotic control approaches, which increase active participation of the patients and allow more kinematic variability while still guaranteeing successful task execution, have the potential to substantially boost the efficacy of robot-aided rehabilitation, especially in functionally more advanced patients. Numerous research groups have been working on these *patient-cooperative *control strategies [24-34]. While there have been extensive tests of control strategies that increase patient participation during training for upper-extremity robots [35,36], most of the approaches for lower extremity-robots have only been evaluated in single case studies with patients or in proof-of-concept experiments with healthy volunteers.

In a recent publication, our group has demonstrated a patient-cooperative control strategy ("Path Control") for the Lokomat which allows free timing of leg movements while ensuring that the spatial kinematics of the legs stay within definable desired limits [37]. We could show that healthy volunteers participated more actively and with more--especially temporal--variability than in a classical, position controlled training mode. Moreover, we were able to modulate the level of activity by an additional supportive "flow" that did not reduce the amount of movement variability when providing more support. We assume that the ability to modulate the level of required activity will be an important feature to adapt the controller to the individual capabilities of patients, particularly of patients transitioning from a non-ambulatory to an ambulatory state during their rehabilitation process. Finally, we evaluated the feasibility of the path control strategy with 15 individuals with chronic incomplete spinal cord injury (iSCI). Assuming a minimal level of voluntary motor control, the patients were able to train with the patient-cooperatively controlled Lokomat.

In the present paper, we have investigated if the short-term effects found for healthy volunteers do also translate to spinal cord injured patients. More specifically, we have posed the following research questions: (1) Does patient-cooperative robot-aided treadmill training lead to more active participation of individuals with iSCI than classical, position-controlled training? (2) Can we deliberately modulate the activity required by the iSCI patient during the training? (3) Can we increase the variability of the iSCI patients' leg movements while still maintaining functional gait?

## Methods

### Gait training robot

Experiments were performed with the gait rehabilitation robot Lokomat. The robot automates body weight-supported treadmill training of patients with locomotor dysfunctions in the lower extremities such as spinal cord injury and hemiplegia after stroke [38]. It comprises two actuated leg orthoses that are attached to the patients' legs. Each orthosis has one linear drive in the hip joint and one in the knee joint to induce flexion and extension movements of hip and knee in the sagittal plane. Knee and hip joint torques can be determined from force sensors between actuators and orthosis. Passive foot lifters can be added to induce ankle dorsiflexion during swing phase. A body weight support system with a harness attached to the patients' trunk reduces the effective body weight by a definable amount.

### Control algorithms

#### Position control

The first approach implemented for the Lokomat was position control [38]. In this approach, the control algorithm tries to match the pre-defined reference trajectory **q**_ref_(*t*) as closely as possible^1^.

#### Impedance control

A first step towards patient-cooperative behavior of the robot was the implementation of an impedance control algorithm [26]. The actual joint positions **q**_act _are virtually coupled to the reference positions **q**_ref_(*t*) by a simulated spring and damper system with spring stiffness **K **and damping constant **B**. If Δ**q **denotes the control deviation,

(1) $$\Delta q=q_{\text{ref}}\left(t\right)-q_{\text{act}},$$

the desired joint torques ***τ***_c _for the robot drives are

(2) $$\tau_{\text{c}}=K\Delta q+B\frac{\text{d}}{\text{d}t}\Delta q=\left(\begin{array}{cc} K_{\text{hip}} & 0\\ 0 & K_{\text{knee}} \end{array}\right)\Delta q+\left(\begin{array}{cc} B_{\text{hip}} & 0\\ 0 & B_{\text{knee}} \end{array}\right)\frac{\text{d}}{\text{d}t}\Delta q.$$

**Figure 1:**
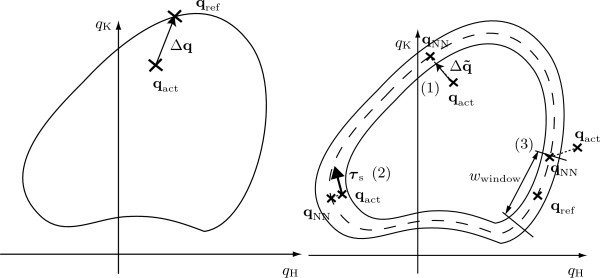
**Control algorithms**. Control algorithms. Impedance control (with its special case position control) is illustrated on the left side. Path control is illustrated on the right side: (1) control action to bring the patient's leg back to the inside of the virtual tunnel, (2) "flow" of supportive torques, (3) "moving window" around time-dependent reference.

By adjusting the parameters of the virtual impedance, the therapist can make the training more or less demanding for the patient. With a very low virtual stiffness, the patient has to participate more actively to maintain a functional gait pattern. In practice, only **K **is adjusted by therapist, and **B **is adapted automatically as a function of **K **[26]. The classical position control mode is included as a special case with **K **set to the maximally achievable stiffness (Fig. 1, left side).

#### Path control

A prominent feature of the position and impedance control approaches is the direct coupling of temporal and spatial guidance. The path control strategy [37] and related approaches [35,39,40] overcome this limitation by providing a virtual tunnel. Within this tunnel, patients can move their legs with their own desired timing of movements. The boundaries of the virtual tunnel provide spatial guidance to make sure that the movements still follow a physiologically meaningful pattern in space.

Details about the implementation of the path control strategy for the Lokomat are given in [37]. In the context of the impedance control algorithm described above, the time-dependent reference **q**_ref_(*t*) is replaced by the nearest neighbor **q**_NN_(**q**_act_) on the spatial pattern template. The modified control deviation $\Delta \overset{ƒ}{q}$ is then the difference between **q**_NN _and **q**_act_, reduced by a dead zone around the tunnel center (Fig. 1, right side, (1)). The spring stiffness rendering the tunnel wall is linearly scaled from zero at the tunnel border to a maximum of *K*_hip _= 720 ^Nm^*/*rad, *K*_knee _= 540 ^Nm^/rad.

For the supporting "flow", a torque vector is calculated by differentiating the reference trajectory **q**_ref _with respect to the relative position in the gait cycle *S*. Thus, the direction of the torque vector is tangential to the movement path in joint space (Fig. 1, right side, (2)).

(3) $${\overset{ƒ}{\tau }}_{\text{s}}(S)=\frac{\frac{\text{d}}{\text{d}S}q_{\text{ref}}(S)}{\left\|\frac{\text{d}}{\text{d}S}q_{\text{ref}}(S)\right\|}$$

The actual supportive torques are

(4) $$\begin{array}{cc} \tau_{\text{s}}(S)={\overset{ƒ}{\tau }}_{\text{s}}(S)\cdot (1-d_{\text{c}})\cdot k_{\text{s}},\; & d_{\text{c}}\in [0,1] \end{array}$$

where *k*_s _is a scalar factor that determines the amount of support in Nm, and *d*_c _is the relative distance of the current position **q**_act _to the center of the path. The relative distance *d*_c _is normalized to the width of the tunnel and saturated to the upper limit 1 for positions **q**_act _outside the tunnel. Thus, supportive torques are only provided within the tunnel.

Finally, a "moving window" can limit free timing to a definable range *w*_window _around the timed reference **q**_ref_(*t*) as it is used by the impedance controller. **q**_NN _is then constrained to be maximally a definable percentage of the gait cycle ahead or behind the timed reference **q**_ref_(*t*) (Fig. 1, right side, (3)).

### Experimental design

Fifteen patients with chronic iSCI (Table 1) participated in a test training session to evaluate if they were able to train successfully with patient-cooperative controllers. Two out of these 15 patients were not able to train with the path control strategy because they had very weak control over their extensor muscles. Hence, they were not able to induce sufficient knee extension at the end of swing phase to move along the desired path. Two other patients dropped out because of personal reasons. The 11 remaining patients volunteered to participate in further experiments.

**Table 1:** Patient characteristics

**Subj**. **No**.	Sex	Age (y)	Lev. of injury	AIS	SCIM (mob.)	WISCI (mob.)	***k***_**s**_ (Nm)
P1	m	31	L2	A	11	12	n/a
P2	m	42	L2	D	18	19	n/a
P3	m	63	L4	D	26	20	5
P4	f	63	Th9	D	29	20	5
P5	f	41	Th9	C	27	18	6
P6	m	63	L3	B	10	16	6
P7	m	51	Th9	C	10	5	7
P8	m	35	C7	D	23	20	5
P9	m	33	L3	B	23	18	6
P10	f	62	L3	D	27	20	4
P11	m	53	L4	A	11	16	n/a
P12	f	64	L3	C	15	16	6
P13	m	31	L1	C	14	12	5
P14	f	53	L3	D	15	20	n/a
P15	m	61	C4	D	17	15	2

iSCI patients were classified according to the ASIA Impairment Scale (AIS) [58]. The capabilities of the iSCI patients were assessed with the mobility subscore of the SCIM III questionnaire [59], which can range from 0 to 30, and with the WISCI II score [60], which can range from 0 to 20. For both scores, higher values indicate better mobility. Patients P1 and P2 were not able to train with the patient-cooperative controller, patients P11 and P14 dropped out because of personal reasons.

All experimental procedures were approved by the Ethics Committee of the Canton of Zurich, Switzerland, and all participants provided informed consent before the experiments.

The 11 chronic iSCI patients trained with the Lokomat at a walking speed of 2 ^km^/_h _(0.55 ^m^/_s_) and with 30-50% body weight support under four different conditions:

1. POS: Position control with the stiffness of the Lokomat controller set to *K*_hip _= 1200 ^Nm^/rad, *K*_knee _= 900 ^Nm^/_rad_^2^.

2. SOFT: Impedance control with the stiffness set to *K*_hip _= 192 ^Nm^/rad, *K*_knee _= 144 ^Nm^/_rad_

3. COOP: Path control with *w*_window _set to 20% of the gait cycle and the support gain *k*_s _adjusted individually for each patient^3^

4. COOP+: Path control with *w*_window _set to 20% of the gait cycle and the support gain *k*_s _increased to 130% of the value used in the previous condition

Prior to the experiment, surface EMG electrodes were attached to the patients' gastrocnemius medialis (GM), tibialis anterior (TA), vastus medialis (VM), rectus femorisi (RF), and biceps femoris (BF) muscles of the left leg. The electrodes were placed according to the SENIAM guidelines [42]. Custom-built foot-switches were taped under the heel of the left foot of the patients to determine heel strikes.

Two additional surface electrodes were placed over the electrical dipole axis of the heart, one below the right clavicle and one below the left pectoral muscle to record a simplified ECG for heart rate extraction. Before each condition, the patients were quietly standing in the Lokomat for 60 seconds. During the final 30 seconds of this period, ECG was recorded to determine the heart rate prior to each condition. After these resting period, patients walked for two minutes to get used to the respective controller. Afterwards, data was recorded during one minute of walking. In addition to the EMG and ECG signals, joint angles from the left hip and knee joints were recorded by sensors at the joint axes of the Lokomat.

### Data analysis

#### Spatiotemporal variability

To quantify the amount of temporal and spatial variations in the gait patterns during walking in the different conditions, we computed the spatio-temporal characteristics of the recorded trajectories **q**_act_(*t*) according to the procedure described by Ilg et al. [43].

The recorded joint angles of each condition were cut into single strides triggered by the heel strike signal of the foot switches. The single strides were normalized in time to the interval [0, 1), with S denoting the normalized stride time. The trajectory of the *k*^th ^normalized stride is referred to as **q**^(*k*) ^(*S*), and the number of recorded strides is denoted *N*. The average trajectory **q**_avg_(*S*) was determined as a reference for the spatio-temporal analysis:

(5) $$q_{\text{avg}}(S)=\frac{1}{N}\sum_{k=1}^{N}q^{\left(k\right)}(S)$$

Each trajectory **q**^(*k*) ^was mapped to the reference trajectory **q**_avg _by a spatial shift function ***ξ***^(*k*) ^(*S*) and a time shift function $\tau_{\text{shift}}^{\left(k\right)}(S)$.

(6) $$q^{\left(k\right)}(S)=q_{\text{avg}}\left(S+\tau_{\text{shift}}^{\left(k\right)}(S)\right)+\xi^{\left(k\right)}(S)$$

The values of the shift functions ***ξ***^(*k*) ^(*S*) and $\tau_{\text{shift}}^{\left(k\right)}(S)$ were determined by optimization as described in [44].

The weighting factor for the optimization was determined according to the rules suggested in [43].

Finally, the spatial variability var_*ξ *_and the temporal variability var_*τ *_as defined in [43] were computed using the following equations:

(7) $${\text{var}}_{\xi }=\frac{1}{N}\sum_{k=1}^{N}\left(\int_{0}^{1}|\xi^{\left(k\right)}(S)|dS\right)$$

(8) $${\text{var}}_{\tau }=\frac{1}{N}\sum_{k=1}^{N}\left(\int_{0}^{1}|\tau_{\text{shift}}^{\left(k\right)}(S)|dS\right)$$

The resulting spatial and temporal variability were compared by a Friedman test (nonparametric equivalent to a repeated measures ANOVA) at the 5% significance level [45]. Multiple comparisons were accounted for by the Bonferroni adjustment.

#### Interaction torques

To better understand the interactions between robot and patient, the interaction torques in the joints of the robot have been calculated. The robot's force sensors are located between drives and exoskeleton and not directly at the interaction points with the human, such that a model of the exoskeleton's dynamics has to be used to derive the interaction torques ***τ***_int _from the torques ***τ***_mot_, which are measured at the robot's drives:

(9) $$\tau_{\text{int}}=\tau_{\text{mot}}-M_{\text{exo}}(q_{\text{act}})\;{\overset{¦}{q}}_{\text{act}}+n_{\text{exo}}(q_{\text{act}},{\dot{q}}_{\text{act}})$$

with **M**_exo _being the mass matrix capturing the inertia of the Lokomat exoskeleton and **n**_exo _subsuming the gravitational, friction, and Coriolis torques of the exoskeleton. Static friction in the joints has been identified in a separate experiment to be below 0.5 Nm and has thus been neglected in the dynamic model. To allow comparisons of the interaction torques under the different conditions, we computed the root mean square over whole recording time *T*_rec_:

(10) $${\overset{ª}{\tau }}_{\text{int}}=\sqrt{\frac{1}{T_{\text{rec}}}\int_{0}^{T_{\text{rec}}}{\left(\tau_{\text{int}}(t)\right)}^{2}\text{d}t.}$$

The root mean square values under the different conditions were compared by a Friedman test (nonparametric equivalent to a repeated measures ANOVA) at the 5% significance level with Bonferroni adjustment.

#### Heart rate

Heart rate was extracted from the simplified ECG recordings by custom Matlab code which determined the length of the RR intervals *I*_RR_. The reciprocal of the median of all RR intervals during the 30 seconds prior to each condition constitutes the pre-condition heart rate

(11) $${\text{HR}}_{\text{pre}}=\frac{\text{l}}{\underset{\text{pre}}{\text{median}}(I_{\text{RR}})}.$$

Analogously, the heart rate during a condition HR_during _was defined as the reciprocal of the median of all RR intervals during the last 30 seconds of each condition. The absolute heart rate increase ΔHR for each condition was then defined as

(12) $$\Delta \text{HR}={\text{HR}}_{\text{during}}-{\text{HR}}_{\text{pre}}.$$

We defined the maximal heart rate increase ΔHR_max _for a specific patient as the maximum of the values for ΔHR under the four different training conditions. Finally, we normalized the absolute heart rate increase for the different conditions with respect to ΔHR_max _to account for the variable cardiovascular reactions of the different patients. The normalization results in the relative heart rate increase Δ HR_rel_

(13) $$\Delta {\text{HR}}_{\text{rel}}=\frac{\Delta \text{HR}}{\Delta {\text{HR}}_{\text{max}}}.$$

The values for ΔHR_rel _under the different conditions were compared by a Friedman test (nonparametric equivalent to a repeated measures ANOVA) at the 5% significance level with Tukey-Kramer adjustment.

#### Muscle activity

EMG signals were band-pass filtered between 15 and 300 Hz, rectified, and cut into single strides triggered by the heel strike signal of the foot switches. The single strides were normalized in time to 1001 samples each. All strides of a patient under a given condition were then averaged. Next, the average strides were broken up into seven phases (initial loading, mid stance, terminal stance, pre-swing, initial swing, mid swing, terminal swing) according to Perry [46]. The root mean square (RMS) of the EMG signals was calculated for each muscle within each of these phases.

The RMS values of the EMG signals showed high inter-subject variability, and the repeated measurements for a single subject were not independent of each other. Linear mixed models [47] are a statistical tool that can account for such circumstances. In these models, random variables can capture the covariance of multiple data values originating from different individual sources. The remaining subject-independent effects can be described as the linear influence of fixed factors.

To investigate the influence of the different conditions on muscle activity, we fitted a separate linear mixed model to the logarithm of the RMS values of the EMG signals of each muscle. For a given muscle, we define the logarithmized RMS for an observation *j *in a subject *i *as EMG*_ij _*. An observation is a combination of one of the four conditions and one of the seven gait phases. Hence, there were 7 × 4 = 28 observations *j *(*j *= 1, 2, ..., 28) per subject. We included the factors "condition" and "gait phase" as fixed effects. Thus, the value of EMG*_ij _*for a given observation *j *on the *i*-th subject was modeled as

(14) $$\begin{array}{c} {\text{EMG}}_{ij}=\beta_{0}+\beta_{1}\times \text{COND}1_{ij}+\beta_{2}\times \text{COND}2_{ij}\\ +\beta_{3}\times \text{COND}3_{ij}+\beta_{4}\times \text{PHASE}1_{ij}\\ +\beta_{5}\times \text{PHASE}2_{ij}+...+\beta_{9}\times \text{PHASE}6_{ij}\\ +u_{0i}+\varepsilon_{ij}. \end{array}$$

The indicator variables COND1*_ij _*to PHASE6*_ij _*were set to one, if the observation *j *belonged to the respective condition or gait phase, otherwise to zero. To account for the correlation of repeated measurements within a subject *i*, a random intercept *u_0i _*was assumed for each subject. The residual *ε_ij _*captures the difference between the measured value EMG*_ij _*and the prediction of the model.

In order to compare the different conditions, we computed the estimated marginal means for each condition by averaging the model predictions across the different gait phases. These estimated marginal means were then compared with post-hoc tests at the 5% significance level. In these tests, multiple comparisons were accounted for by the Bonferroni adjustment. A similar statistical analysis of EMG data has been performed in [37] and in [30].

## Results

### Kinematics and spatiotemporal variability

**Figure 2:**
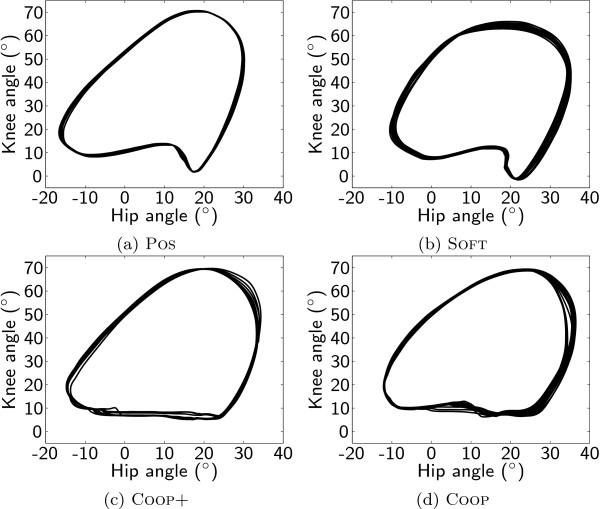
**Kinematic data**. Resulting kinematic data. Trajectories in joint space for one exemplary patient (P12) under the different conditions POS (a), SOFT (b), COOP+ (c), COOP (d).

Patients changed their gait kinematics notably under the different training conditions (Fig. 2). The virtual tunnel in the path control modes allowed for a less extended knee at initial contact, and consequently, patients reduced their peak knee extension. Patients also increased their maximal hip flexion during swing phase in the path control modes.

**Figure 3:**
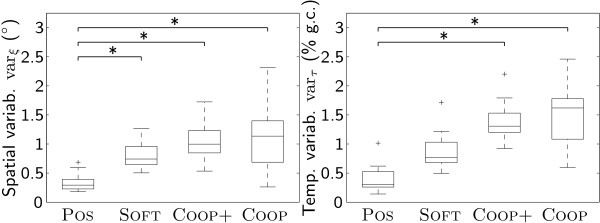
**Spatiotemporal variability**. Spatial variabilty var_*ξ *_(°) and temporal variability var_*τ *_(% gait cycle).

Spatial variability under conditions SOFT (soft impedance control mode), COOP (path control mode), and COOP+ (path control mode with increased supportive flow) was significantly higher than under condition POS (stiff position control mode). There were no significant differences between the conditions SOFT, COOP+, and COOP (Fig. 3, left).

Temporal variability under the conditions COOP+ and COOP was significantly higher than under condition POS. Condition SOFT was not significantly different from any other condition (Fig. 3, right).

### Interaction torques

**Figure 4:**
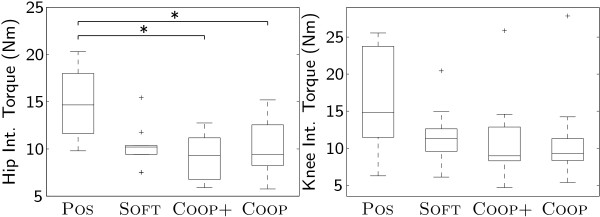
**Interaction torques**. Interaction torques ***τ***_int _(Nm) for hip and knee joint.

Interaction torques in the hip joint between patient and robot were significantly smaller under conditions COOP+ and COOP than under condition POS. No significant differences between the conditions could be found for the interaction torques in the knee joint (Fig. 4).

### Heart rate

**Figure 5:**
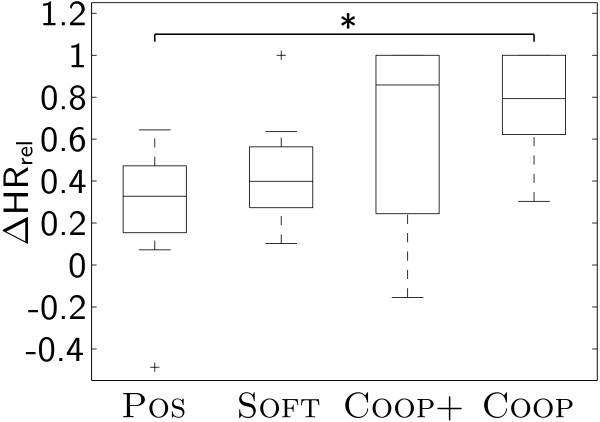
**Relative change of heart rate**. Relative change of heart rate ΔHR_rel _while walking under the different conditions.

The relative heart rate increase ΔHR_rel _was significantly larger under condition COOP than under condition POS. No other significant differences could be identified (Fig. 5).

### Muscle activity

**Figure 6:**
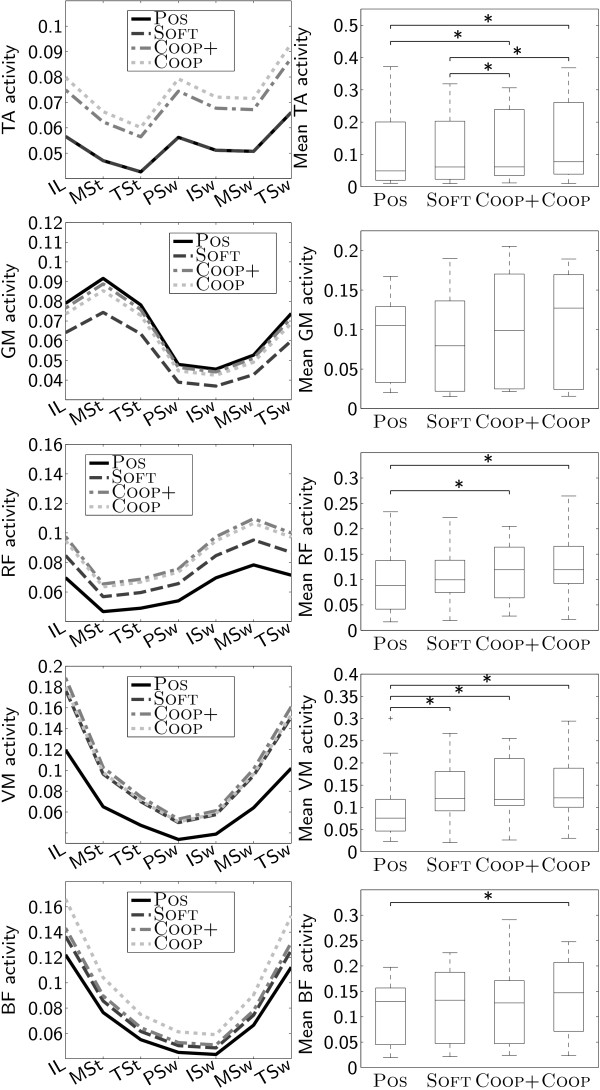
**Muscle activity**. Muscle activity of TA (Tibialis anterior), GM (Gastrocnemius medialis), VM (Vastus medialis), RF (Rectus femoris), and BF (Biceps femoris) muscles as predicted by the linear mixed models (left column). Comparison of mean muscle activity under the different conditions (right column).

Activity of the Tibialis anterior muscle was significantly increased under the COOP+ and COOP conditions compared to the POS and the SOFT conditions. No significant differences could be found for the activity of the Gastrocnemius medialis muscle. Activity of the Rectus femoris muscle was significantly increased under the COOP+ and COOP conditions compared to the POS condition. For the Vastus medialis muscle, conditions SOFT, COOP+, and COOP caused significantly higher activity than POS. Activity of the Biceps femoris muscle was significantly higher under the COOP condition than under the POS condition (Fig. 6).

## Discussion

### Active participation

Basic neuroscience studies have shown that motor learning is more effective when human subjects practice movements actively rather than being passively moved [18,19,48,49]. Although the underlying mechanisms are not well understood yet, this principle is generally translated also to robotic neurorehabilitation [23], where researchers aim at making patients participate as actively as possible during training.

Our evaluation has shown that iSCI patients participated with higher muscle activity (Fig. 6) and higher cardiovascular effort (increased heart rate, Fig. 5) when they were training under the path control condition (COOP) than under the position control condition (POS). Theoretically, this increased activity could also be caused by the robot generating torques opposed to the movements of the patient. While there are studies investigating the effects of such robotic resistance [50], our goal was to obtain active, unobstructed participation of the patients. The fact that interaction torques did not increase under the path control conditions (Fig. 4) shows that the patients were indeed contributing actively to the movements and not working against robotic resistance.

We have included a condition with soft impedance control (SOFT) as a benchmark for the current state-of-the-art of patient-cooperative Lokomat training in clinical practice. The impedance setting (*K*_hip _= 192 ^Nm^/_rad_, *K*_knee _= 144 ^Nm^/_rad_, these values correspond to a "guidance force" setting of 40% in the commercial Lokomat software) for this condition was chosen based on discussions with the physical therapy staff at University Hospital Balgrist (Zurich, Switzerland) about the lowest impedance settings they use during clinical trainings on a regular basis. Interestingly, it appears that the remaining temporal guidance (Fig. 3, right) in this compliant control mode still kept the patients in a rather passive state: Only the vastus medialis muscle was significantly more active in compliant control mode than in position control mode. All other parameters did not differ significantly (Fig. 5, Fig. 6). This observation is in line with theoretical models of human-robot interactions which predict that the human motor system will "slack" whenever possible to reduce its effort [51-54]. Apparently, the free timing of movements provided by the path control strategy which requires patients to actively propel their legs through the gait pattern makes patients less likely to "slack" than the timing-based soft impedance control mode used under condition SOFT.

Thus, the iSCI patients in our experiment participated more actively during training only with the patient-cooperative path control strategy.

### Modulation of activity by additional support

Unlike in our study with healthy volunteers [37], we were not able to modulate activity by adjusting the amount of additional support. Apparently, subjects reacted very inconsistently to the increased support in condition COOP+. While for some subjects the additional support was actually helpful, others felt "pushed forward" and had to put more effort in actively canceling this "perturbation". This effect may be the reason for the large variability of heart rate increase under the condition COOP+ (Fig. 5).

As already seen in the feasibility experiment with iSCI subjects in [37], iSCI patients have diverse needs for support, usually limited to specific gait phases. Therefore, the "global" support parameter *k*_s _which determines the intensity of the supportive "flow" for the whole gait cycle appears to be not sufficient to adapt the support for iSCI patients. For an impedance controller based on a reference pattern with fixed timing, gait-phase dependent adaptation of controller impedance has been demonstrated by Emken et al. [33]. For the path control strategy evaluated in this paper, which allows free timing of movements, an automatic adaptation algorithm that identifies the individual deficits of a patient as implemented for the upper extremity by Wolbrecht et al. [55] could possibly improve the training mode by providing support that is better tailored to the individual patients.

### Movement variability

Variability and the possibility to make errors is considered an essential component of practice for motor learning. Bernstein's demand that training should be "repetition without repetition" [21] is still considered a crucial requirement, which is also supported by recent advances in computational models describing motor learning [23]. More specifically, a recent study by Lewek et al. [22] has shown that intralimb coordination after stroke was improved by manual training after stroke, which allowed kinematic variability, but not by position-controlled Lokomat training, which reduced kinematic variability to a minimum.

The analysis of spatiotemporal variability shows that while spatial variability is significantly increased in all three compliant modes SOFT, COOP+, and COOP compared to the stiff position control condition POS, temporal variability is only significantly increased in the path control modes COOP+ and COOP.

The virtual tunnel of the path control strategy allowed spatial variability to an extent that still ensured a functional gait pattern, therefore, it did not substantially increase the patients' risk of stumbling.

Thus, the path control strategy does not only technically provide free timing of movements, but iSCI patients also showed more temporal variability in their movements than with position control (POS) or with the compliant, but timing-controlled impedance control (SOFT).

### Limitations

#### Limitations of the path control strategy

It should be noted that a constant treadmill speed was used throughout the presented experiments. Thus, the temporal freedom of the path control mode were limited to the swing phase. Nevertheless, a substantial increase in temporal variability could be detected. To increase patient interactivity during training, we will combine the path control strategy with approaches which adapt the treadmill speed according to the intention of patients [56].

The fixed walking pattern that defines the spatial movement path may not be ideal for every patient. As in position-controlled Lokomat training, the pattern can be adapted manually by the therapist. However, it is not guaranteed that a pattern close to the "healthy" pattern of an individual patient can be achieved. For hemiparetic patients, it would be possible to derive a desired path for the affected leg from observing the unaffected leg, as proposed by Vallery et al. [32]. For iSCI patients, an adaptive re-shaping of the path, similar to the approach by Jezernik et al. [25], may improve the applicability of the path control strategy.

#### Limitations of the study

The present study only investigated the reactions of iSCI patients to different controllers during a single training session with short exposure to the different training modes. Clearly, the long term effects of the different training modes are much more important and should be investigated in future work. However, we believe that verifying the intended, presumably beneficial effects in a single training session was an important first step in preparation of a long term trial.

We deliberately included patients with a wide range of ambulatory skills to gain insights into the feasibility of path control training with patients at different skill levels. The distribution of walking skills comprised four fully ambulatory patients with a WISCI score of 20, indicating that they were able to independently ambulate 10 m without any walking aids. Furthermore, six patients had reduced, but good ambulatory skills (WISCI score between 12 and 19) and were able to independently ambulate 10 m using appropriate walking aids (crutches and braces). Finally, there was one patient in the transition range between non-ambulatory and ambulatory, indicated by a WISCI score of 5. As we expect the most practical benefits of patient-cooperative control strategies for patients in the transition range between non-ambulatory and ambulatory, more data regarding the feasibility with functionally more restricted patients would be desirable. Thus, future studies with the path control strategy should more explicitly focus on patients within this functional range.

As we planned to include patients with very different walking skills, we decided that it would have been very difficult to reliably standardize a control condition where patients would have walked without assistance or manual assistance of a therapist. Therefore, we performed our experiments without such a condition which would of course have allowed very interesting further analyses. Future studies which will be focusing on patients from a more narrow functional range. As these patients will have similar--and thus standardizable--needs for support during manual assisted treadmill training, it will then be feasible to include such a condition.

The limited number of patients included in the study does not provide sufficient statistical power to stratify patients according to their disability levels, which might reduce the variability in the results and provide further insights into the different effects of the evaluated control strategies on different groups of patients. The focus of the study on iSCI patients leaves it an open question whether similar results can be expected for patients with stroke or other pathologies. The feasibility of patient-cooperative training and the immediate effects for such patients needs to be investigated separately.

The choice of heart rate as a measure of effort was made because it did put a relatively low additional burden on the patients during the experiment. As discussed by Pennycott et al., heart rate may be influenced by emotional state, pain and hydration level, whereas oxygen uptake would be a more robust measure of effort during robot-aided gait training [57]. These factors may explain the large variability under the condition COOP+ where some patients may have been irritated by the increased amount of robotic support. However, as the general trend of the heart rate results is consistent with the results regarding the muscle activity of the patients, we believe that the method has captured the patients' effort in a sufficiently robust way for the sake of our research questions.

## Conclusions

Patients with incomplete spinal cord injury participated more actively and with larger kinematic variability in patient-cooperative robot-aided gait training than in non-cooperative, position-controlled robot-aided gait training. Free timing of movements appears to be an important feature of patient-cooperativeness, as a compliant impedance control mode with fixed timing did not significantly increase active participation, but the path control strategy with free timing did.

Future development should focus on providing adaptive, patient-specific support to make training with patient-cooperative control strategies feasible for a larger population of patients. Future clinical evaluation should compare the effects of patient-cooperative robot-aided training versus non-cooperative robot-aided training and manual BWSTT in a long term randomized clinical trial.

## Authors' contributions

AD and AC contributed equally to this work. AD and AC performed the measurements of all patients, data analysis, statistical analysis, and drafted the manuscript. RR participated in the design and coordination of the study and assisted with drafting the manuscript. All authors read and approved the final manuscript.

## Foot Notes

^1^The following notation is used throughout this paper: all vectors of joint angles and torques consist of two elements, the first one for the hip joint and the second one for the knee joint, e.g. **q **= (*q*^(1)^, *q*^(2)^)^T ^= (*q*_hip_, *q*_knee_)^T^. The control algorithms discussed in this paper are always defined for a single leg. The second leg is controlled by an independent second instance of the respective control algorithm.

^2^The equivalent end-point stiffness of the exoskeleton depends on the joint angles and the direction of force application and, thus, can not be reflected in a single, representative number. The relationship between end-point stiffness and joint stiffness in a lower-limb exoskeleton is discussed in [41].

^3^The therapist was instructed to adjust *k*_s _to the minimal value that enabled the patient to walk in the path control mode. The individual support gains which were used under this condition are listed in Tab. 1.
